# Discrepancy of Breast and Axillary Pathologic Complete Response and Outcomes in Different Subtypes of Node-positive Breast Cancer after Neoadjuvant Chemotherapy

**DOI:** 10.7150/jca.62830

**Published:** 2021-07-06

**Authors:** Shin-Cheh Chen, Chi-Chang Yu, Hsien-Kun Chang, Yung-Chang Lin, Yung-Feng Lo, Shih-Che Shen, Wen-Lin Kuo, Hsiu-Pei Tsai, Hsu-Huan Chou, Chia-Hui Chu, Wen-Chi Shen, Ren-Chin Wu, Shir-Hwa Ueng, Yi-Ting Huang

**Affiliations:** 1 Department of General Surgery, Chang Gung Memorial Hospital at Linkou, Chang Gung University College of Medicine, Taoyuan, Taiwan; 2 Department of Hematology-Oncology, Chang Gung Memorial Hospital at Linkou, Chang Gung University College of Medicine, Taoyuan, Taiwan; 3 Department of Pathology, Chang Gung Memorial Hospital at Linkou, Chang Gung University College of Medicine, Taoyuan, Taiwan; 4 Department of Radiation Oncology, Chang Gung Memorial Hospital at Linkou, Chang Gung University Medical College, Taoyuan, Taiwan

**Keywords:** breast cancer, neoadjuvant chemotherapy, pathologic complete response, intrinsic subtype

## Abstract

Few studies have analyzed the discrepancy between breast pathologic complete response (B-pCR) and axillary node pCR (N-pCR) rates and their impact on survival outcomes in different intrinsic subtypes of early breast cancer after neoadjuvant chemotherapy (NAC). We retrospectively reviewed B-pCR, N-pCR, and total (breast and axillary node) pCR (T-pCR) after NAC to assess the discrepancy and outcomes between 2005 and 2017. A total of 968 patients diagnosed with cT1-4c, N1-2, and M0 breast cancer were enrolled in the study. The median age was 49 years and the median follow-up time was 45 months. Of these patients, 213 achieved T-pCR, 31 achieved B-pCR with axillary node pathologic non-complete response (N-non pCR), 245 achieved N-pCR with breast pathologic non-complete response (B-non pCR), and 479 achieved total (breast and axillary node) pathologic non-complete response (T-non pCR) after NAC. The highest B-pCR and N-pCR rates were found in the hormone receptor-negative, human epidermal growth factor receptor 2-positive HR(-)HER2(+) subtype, while the lowest B-pCR rate was found in the HR(+)HER2(-) subtype. The N-pCR rate was correlated to the B-pCR rate (P<0.001), but was higher than the B-pCR rate in all subtypes. The 5-year overall survival (OS) rates for patients with T-pCR, B-pCR, and N-pCR were 91.2%, 91.7%, and 91.9%, respectively. For non-pCR, non-pCR, and non-pCR, the 5-year OS rates were 73.6%, 78.9%, and 74.7%, respectively (P<0.0001). B-non pCR patients had a lower risk of recurrence than T-non pCR or N-non-pCR patients, although there were no differences in OS among them. In conclusion, the N-pCR rate was higher than the B-pCR rate after NAC in all intrinsic subtypes, and N-non pCR or T-non pCR patients had the worst outcomes.

## Introduction

Neoadjuvant chemotherapy (NAC) is a treatment strategy for increasing the rate of breast-conserving surgery in operable breast cancer and increasing operability in inoperable breast cancer, especially in triple-negative and human epidermal growth factor receptor 2 (HER2) overexpression subtypes. Pathologic complete response (pCR) rates vary among different subtypes but are considered the most important surrogate markers of disease-free survival (DFS) and overall survival (OS). However, some contradictory meta-analyses have been reported.^1^-^7^ High pCRs of the breast tumor itself (ypT0/is, B-pCR) and axillary lymph nodes (ypN0, N-pCR) have been proposed to be linked to the omission of primary surgery after minimally invasive biopsy 8 and de-escalation axillary dissection by sentinel node biopsy.^9^

The discrepancy between B-pCR and N-pCR rates in clinically node-positive (cN+) disease has rarely been reported, except for tumor heterogeneity as the underlying biological event to explain the difference in primary disease and metastatic nodes.^10^ N-pCR rates were reported to be higher than B-pCR rates in cN+ patients, although inconsistent results of N-pCR rates have been reported in various intrinsic subtypes of cN+ disease.^7^, ^11^, ^12^ There are many predictive models to identify N-pCR for omission of axillary surgery, although this is not practical.^13^-^15^

Moreover, only a few studies have discussed the impact of B-pCR or N-pCR separately on survival benefit after NAC 7, 8, 13, 15 and there are only a limited number of trials that have defined the impact of non-pCR either in the breast or axillary node on survival.^16^ Nevertheless, residual nodal disease is considered a more important prognostic factor of survival than residual breast burden. 11

In this study, we analyzed the differences between total (breast and axillary node) pCR (T-pCR), B-pCR, and N-pCR rates in cN+ breast cancer after NAC in various intrinsic subtypes. Further, exploring the role of B-pCR and/or N-pCR contributes to the survival benefit and impact of non-pCR either in the breast or axilla nodes on outcomes in different intrinsic subtypes.

## Material and methods

We conducted a retrospective review of all breast cancer patients treated with NAC between January 2005 and December 2017 at our institution. The inclusion criteria consisted of women aged 20 years or older who (1) had histologically proven clinical stage T1 through T4c, N1 through N2, M0 primary invasive breast cancer according to the eight edition of the American Joint Commission on Cancer TNM staging system, (2) had axillary nodal disease confirmed by fine-needle aspiration or core-needle biopsy, and (3) had underwent curative surgery after NAC. The exclusion criteria consisted of patients who (1) had synchronous bilateral breast cancer, (2) had clinically node-negative (cN0) status, (3) had inflammatory breast cancer, and (4) had distant metastases. In our institution, axillary nodal status was routinely determined before NAC administration using axillary ultrasound. If axillary ultrasound showed no suspicious lymph nodes, the patient was defined as cN0. The patients were defined as cN+, while suspicious lymph nodes were confirmed with additional fine-needle aspiration cytology or core-needle biopsy. cN1 was defined as metastasis to movable ipsilateral level I and/or level II axillary nodes, and cN2 was defined as metastasis to fixed or matted ipsilateral level I and/or level II axillary lymph nodes. The administered NAC regimens included the following: four cycles of TE (docetaxel, epirubicin); or four cycles of fluorouracil, epirubicin, and cyclophosphamide followed by four cycles of docetaxel. In the case of HER2(+) breast cancer, trastuzumab was recommended as targeted therapy in addition to chemotherapy and continued for 1 year. No HER2-targeted therapy was advised from 2005-2010.

HER2 status was evaluated by immunohistochemistry (IHC). An IHC score of 0 or 1+ was considered negative. In the case of a 2+ IHC score, fluorescence in situ hybridization (FISH) was mandatory in addition to IHC. An IHC score of 3 (>10% of cells with strong intensity circumferential membrane staining) or FISH positivity was defined as positive. Hormonal receptor (HR) positivity was defined as an IHC score of estrogen receptor or progesterone receptor >1%.

The cohort of patients was categorized into four intrinsic subtypes based on combinations of HR and HER2 status: HR(+)HER2(-), HR(+)HER2(+), HR(-)HER2(+), and HR(-)HER2(-). pCR was defined as the absence of residual invasive carcinoma in the surgical specimen. Response to NAC was categorized as B-pCR (ypT0/is) as no invasive carcinoma in the breast after NAC, N-pCR (ypN0) as no invasive carcinoma in the axillary node after NAC, T-pCR (ypT0/is ypN0) as no invasive carcinoma in both the breast and axillary nodes after NAC, B-non pCR as invasive carcinoma in the breast after NAC, N-non pCR as invasive carcinoma in the axillary node after NAC, and T-non pCR as invasive carcinoma in both the breast and axillary nodes. This study was approved by the Institutional Review Board of Chang Gung Memorial Hospital (IRB/CGMH) in Taiwan (IRB No. 201601335B0).

Chi-square tests and analysis of variance were used to assess the differences in categorical and continuous variables, proportions, and median values with interquartile ranges (IQRs). Six post-hoc tests with Bonferroni correction with α=0.05 were simultaneously performed, and the correction was 0.05/6=0.00833. A logistic regression model was used to estimate the associations between subtypes and post-NAC responses after adjusting for clinicopathologic characteristics. DFS was defined as the time from diagnosis to the date of breast cancer recurrence, the date of death from any cause, or the date of the last follow-up. OS was defined as the time from diagnosis to the date of death or last follow-up. Kaplan-Meier (KM) curves were used to visualize unadjusted OS for the entire cohort, and receptor subtype, with log-rank P<0.05, was defined as significant. Cox proportional hazards modeling was used to estimate the association between subtypes and post-NAC response. The hazard ratios and 95% confidence intervals (95% CIs) with two-tailed P<0.05 were considered significant. All analyses were conducted using SPSS version 20.0 (Statistical Package for the Social Sciences Version 20.0 IBM corp. Armonk, NY).

## Results

A total of 968 patients were included in this analysis. Clinical features are summarized in Table 1. The median age at diagnosis was 49 (IQR: 14.5) years, and the average tumor size was 4.2 (IQR: 2.4) cm. The median follow-up time was 45 (range, 5.1-163.2) months. There were 543 (56.1%) patients categorized as having cN1 disease, and the remaining 425 (43.9%) had cN2 disease. The distribution of intrinsic subtypes according to HR and HER2 status was as follows: 382 (39.5%) patients were HR(+)HER2(-), 222 (22.9%) were HR(+)HER2(+), 216 (22.3%) were HR(-)HER2(+), and 148 (15.3%) were HR(-)HER2(-). Anthracycline and taxanes were included in the treatment regimens for all subtypes, but using different schedules. Cisplatin combined with taxanes was used in 93 (63%) HR(-)HER2(-) patients. Trastuzumab alone was administered to 242 (55.3%) HER2(+) patients and dual blockade with trastuzumab plus pertuzumab was administered to 66 (15.1%) HER2(+) patients.

There were 213 (22.0%) patients who achieved T-pCR, 31 (3.2%) patients achieved B-pCR with N-non pCR, 245 (25.3%) patients achieved N-pCR with B-non-pCR, and 479 (49.5%) patients achieved T-non pCR. In this cohort, we found a significantly higher N-pCR rate (458 patients, 47.3%) than B-pCR (244 patients, 25.2%). The pCR and non-pCR rates according to subtype are shown in Table 2. The lowest B-pCR rate (29 patients, 7.6%) was found in the HR(+) HER2(-) subtype, while the highest B-pCR rate (95 patients, 44.0%) in HR(-)HER2(+) and highest N-pCR (133 patients, 61.6%) were found in the HR(-)HER2(+) subtype. The N-pCR rate was highly correlated with B-pCR (P<0.0001), and even higher rates (the differences: 22.5%-24.8%) were found in all molecular subtypes except HR(-)HER2(+) ( difference: 17.6%). In B-non-pCR patients, the N-pCR rates were 25.2%, 43.0%, 39.7%, and 43.4% for HR(+)HER2(-), HR(+)HER2(+), HR(-)HER2(+), and HR(-)HER2(-) subtypes, respectively. In contrast, in N-non-pCR patients, the B-pCR rates were 10.3%, 14.1%, 10.5%, and 16.3% in HR(+)HER2(-), HR(+)HER(+), HR(-)HER(+), and HR(-)HER2(-) subtypes, respectively. A relatively higher N-pCR rate in B-pCR patients in comparison to B-non pCR was found either in cN1 or cN2 diseases. Very high N-pCR rates (range, 86.1%-92.6%) were found in B-pCR patients with cN1 disease in all subtypes (Supplementary Table 1A, 1B).

**Figure 1:**
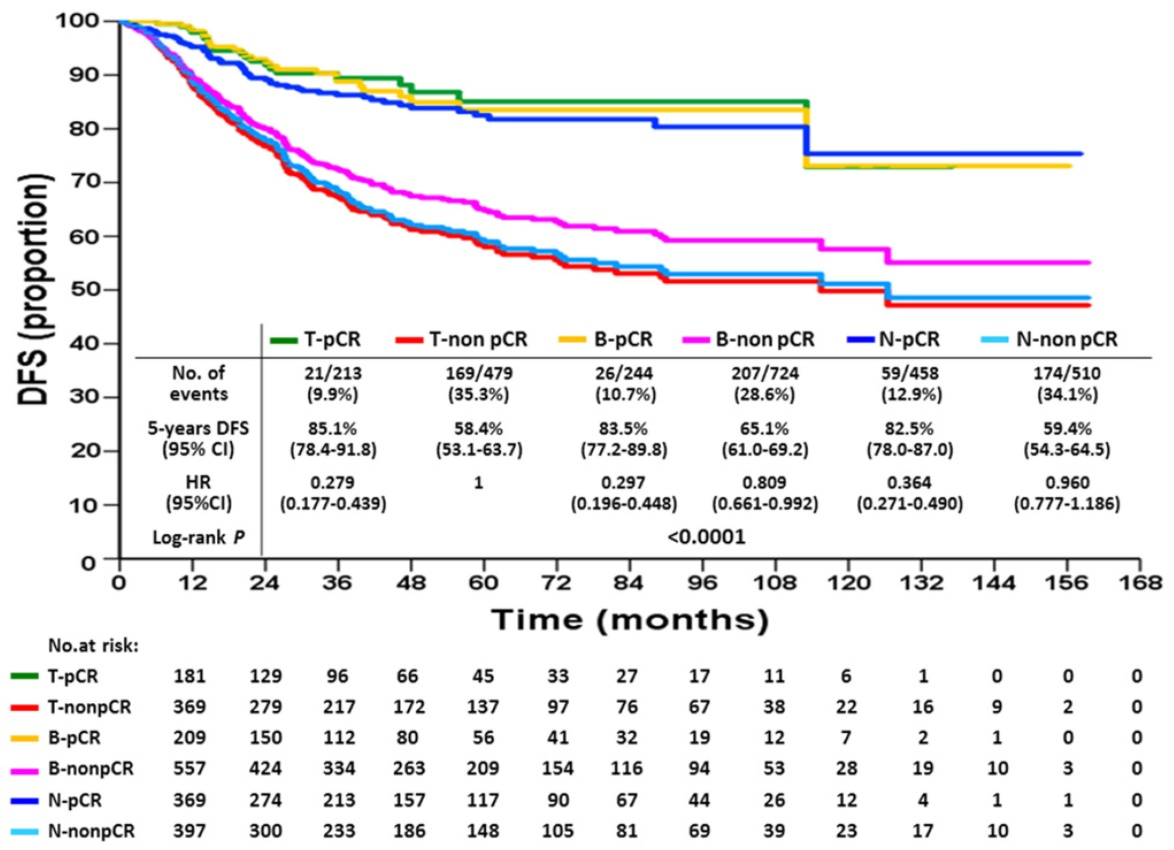
Disease-free survival according to breast or axillary node response status after neoadjuvant chemotherapy

**Figure 2:**
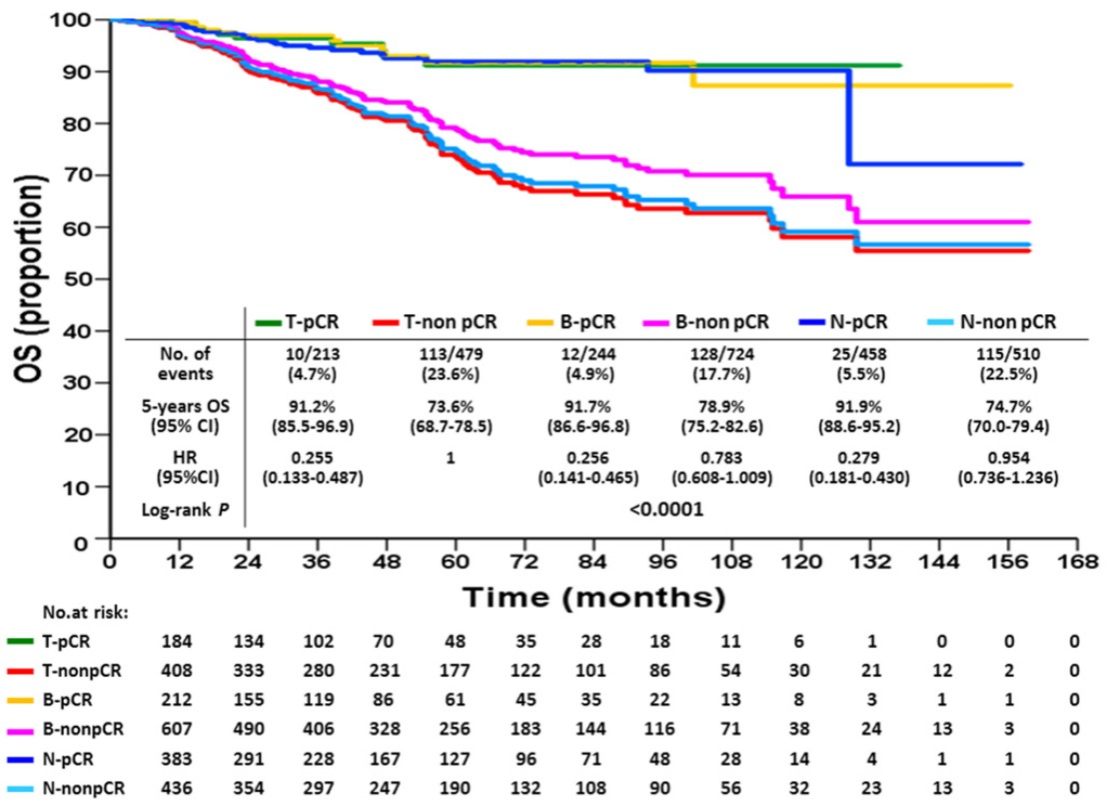
Overall survival according to breast or axillary node response status after neoadjuvant chemotherapy

KM DFS and OS curves according to different response statuses are shown in Figures 1 and 2. The 5-year DFS rates for patients with T-pCR, B-pCR, and N-pCR were 85.1% (95% CI, 78.4%-91.8%), 83.5% (95% CI, 77.2%-89.8%), and 82.5% (95% CI, 78.0%-87.0%), respectively. For T-non pCR, B-non pCR, and N-non pCR, it was 58.4% (95% CI, 53.1%-63.7%), 65.1% (95% CI, 61.0%-69.2%), and 59.4% (95% CI, 54.3%-64.5%), respectively (P<0.0001). The 5-year OS rates for patients with T-pCR, B-pCR, and N-pCR were 91.2% (95% CI, 85.5%-96.9%), 91.7% (95% CI, 86.6%-96.8%), and 91.9% (95% CI, 88.6%-95.2%), respectively. For T-non pCR, B-non pCR, and N-non pCR, the 5-year OS rates were 73.6% (95% CI, 68.7%-78.5%), 78.9% (95% CI, 75.2%-82.6%), and 74.7% (95% CI, 70.0%-79.4%), respectively (P<0.0001).

**Figure 3:**
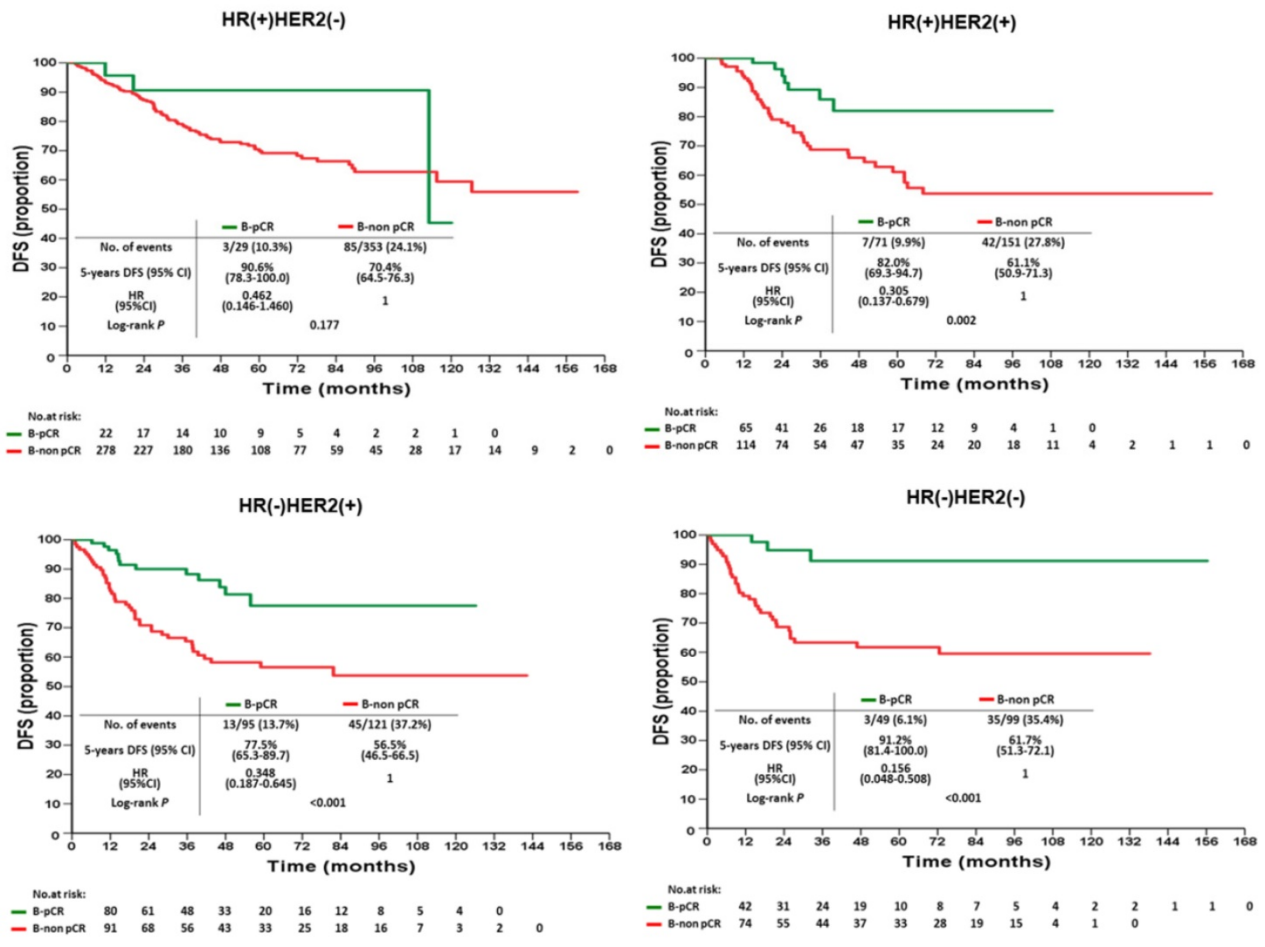
Disease-free survival of B-pCR and B-non pCR patients after neoadjuvant chemotherapy by intrinsic subtypes

**Figure 4:**
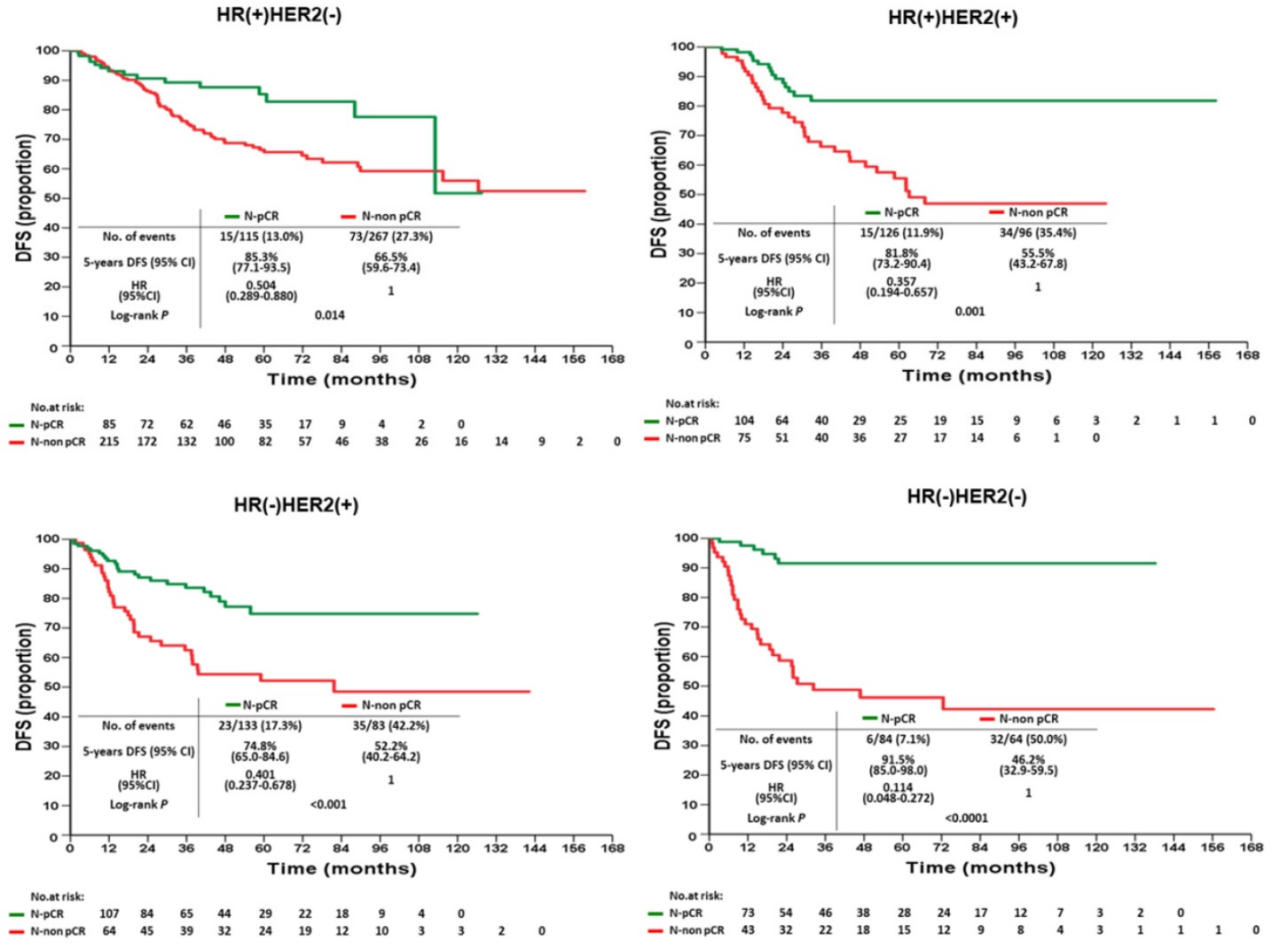
Disease-free survival of N-pCR and N-non pCR patients after neoadjuvant chemotherapy by intrinsic subtypes

According to various intrinsic subtypes, a significantly better 5-year DFS was found in B-pCR patients than in B-non-pCR patients in all intrinsic subtypes except HR(+)HER2(-) (P=0.177, Figure 3). The differences in 5-year OS between B-pCR and B-non pCR in various subtypes are shown in Supplementary Figure 1A. N-pCR patients had significantly better 5-year DFS and 5-year OS than N-non-pCR patients in all subtypes, including the HR(+)HER2(-) subtype (P=0.014 and 0.027, respectively; Figure 4 and Supplementary Figure 1B).

The univariate and multivariate Cox proportional hazards models of DFS, adjusted for response are summarized in Table 3 and for OS in Table 4. With respect to DFS, the following factors were associated with an increased risk of recurrence: higher T stage, cN2 disease, high histologic grade, no hormonal therapy, and non-pCR. For OS, the risk factors of increased death were higher T stage, cN2 disease, high histologic grade, no hormonal therapy, and non-pCR. Compared with T-non pCR, T-pCR patients had a lesser risk of recurrence (hazard ratio 0.35, 95% CI: 0.22-0.56, P<0.0001) and death (hazard ratio 0.37, 95% CI: 0.19-0.72, P=0.001). The same trends were found either in B-pCR or N-pCR patients. Patients with B-non pCR had a lower risk of recurrence (hazard ratio 0.81, 95% CI: 0.66-0.99, P=0.041) than those with non-pCR, although they lost a significant difference on multivariate analysis; further, there was a non-significant trend of favorable OS in B-non pCR than in T-non pCR. The N-non pCR and T-non pCR patients had the worst 5-year DFS and OS rates.

## Discussion

In this retrospective analysis from a single hospital, we demonstrated significantly higher N-pCR rates than B-pCR in all subtypes after NAC, and only a small proportion of N-non-pCR cases (10.3%-16.3%) were found in these patients. In contrast, our study showed very high N-pCR rates (39.7%-43.4%) among B-non-pCR patients in all subtypes except HR(+)HER2(-) (25.2%). The study also confirmed that pCR in the breast, axillary node, or both was associated with improved DFS and OS compared with those with residual cancer. In patients with residual disease, T-non pCR and N-non pCR had worse DFS than B-non-pCR.

Positive axillary lymph node status is a more important prognostic factor than tumor size in early breast cancer and remains the worst prognostic indicator following NAC. There is much evidence suggesting that metastatic lymph nodes need to be treated because cancer cells in lymph nodes can exit and spread to distant metastatic sites. The lymph node window model experiment showed that cancer cells in lymph nodes cause lymphatic vessel regression and lack of angiogenesis in the formation of lymph node metastasis 17, which induces poor control and difficulty in eradicating the disease in metastatic lymph nodes. Inconsistent results and very high alterations in both estrogen receptor and HER2 status were observed throughout the process of tumor progression.^18^ Furthermore, different clone mutations in the axillary node with additional copy number changes were demonstrated in a genomic study using single-cell sequencing 19, and few new mutations were not observed in the primary tumor with key driver mutation in synchronous lymph node metastasis.^20^ These findings indicate difficulty in treating metastatic axillary nodes compared with breast tumors. However, many studies, including ours, have shown very high N-pCR rates (23%-61%).^21^ The paradoxical findings of difficult treatment and good axillary nodal response rate to NAC cannot simply be explained by the biological nature of the disease or genomic findings. The relatively smaller metastatic tumor volume within the axillary node in comparison to the breast tumor itself should be one of the predictive factors. The difference in N-pCR rates between cN1 and cN2 disease in our study confirmed that the difference in tumor size burden of metastatic nodes is one of the contributing factors of N-pCR (Supplementary Table 1A, 1B). Through literatures reviewed, relative low N-pCR rates (around 20%) were reported with conventional chemotherapy 2 decades ago 22, 23, while much higher N-pCR rates after incorporating intensive chemotherapy and targeted therapy were reported in the last decade. 24, 25 Our data and others 26 showed that there were very high N-pCR rates in this cohort. Similarly, high N-pCR rates (25.2%-43.4%) were found even in B-non pCR patients in various intrinsic subtypes. The high N-pCR rates found in B-non-pCR patients have also been poorly explored. Besides the smaller tumor burden in axillary nodes, the observation that the cancericidal effect of concurrent novel agents such as trastuzumab and intensive chemotherapy occurred mainly on metastatic lymph nodes rather than the tumor itself was a possible explanation.

NAC for operable breast cancer can eliminate axillary nodal metastasis to avoid axillary dissection, and several prediction models on this have been developed.^12^, ^27^-^29^ One prospective and another retrospective study found that N-pCR is highly correlated with B-pCR, and nodal positivity rates were <2% after NAC in HER2(+) and triple-negative breast cancer. This provided the rationale for omitting axillary surgery in specific subtypes while B-pCR was achieved.^11^, ^30^ Although high incidence rates (83.7%-89.7%) of N-pCR were found in B-pCR patients in our study, they did not support the hypothesis of de-escalating axillary surgery if B-pCR was confirmed after minimally invasive biopsy, because worse DFS was found in patients with residual axillary node disease after NAC compared with residual disease in the breast. This finding underlines the prognostic relevance of pathologic nodal status after NAC, which is consistent with recent data suggesting that sentinel node biopsy should not be routinely substituted for axillary dissection with ypN1 disease.^31^

Even if the improvement of event-free survival (EFS) could not be established with pCR in clinical trial level analysis 1, many updated studies reported the correlation between pCR and DFS and OS in most subtypes regardless of the chemotherapy regimens administered. 5, 32, 33 A recent meta-analysis including 52 trials also demonstrated that patients with pCR after NAC had significantly better EFS (hazard ratio 0.31, 95% CI: 0.24-0.39) and had associated improved survival (hazard ratio 0.22, 95% CI: 0.15-0.31) except for the HR(+)HER2(-) subtype.^2^ Our study confirmed that patients with pCR, regardless of the breast, axillary node, or both, had better DFS and OS than non-pCR patients in all intrinsic subtypes except HR(+)HER2(-).

Worse outcomes were found in those with residual disease after NAC in most studies, although whether B-non pCR or non-pCR is a more important prognostic factor for poor survival has been rarely studied. Two reports showed that the worst outcomes occurred with N-non-pCR in the HR(-)HER2(-) subtype.^7^, ^19^ In our study, patients with residual disease in either the breast or axillary node had worse outcomes. The worst 5-year DFS was found in non-pCR patients with HR(+)HER2(+), HR(-)HER2(+), and HR(-)HER2(-) subtypes (55.5%, 52.2%, and 46.2%, respectively), and the worst 5-year OS rates were found in the N-non pCR of HR(-)HER2(+) and HR(-)HER2(-)subtypes (64.8% and 56.4%, respectively) (Supplementary Figure 1B). This finding suggests that searching for more effective drugs to eradicate cancer cells in metastatic axillary nodes rather than the breast tumor itself is crucial when designing NAC clinical trials. Therefore, the accurate selection of optimal patients with tailor-appropriate neoadjuvant regimens will improve the N-pCR rate and de-escalate axillary surgery.

As mentioned above, the response of breast tumors or axillary nodes to chemotherapy or novel agents vary. The residual cancer burden (RCB) is one of the standard classifications of the response to NAC, which is based on four parameters of combined response, including breast tumor and axillary node response to conventional chemotherapy regimens.^17^ Our data are consistent with the retrospective studies from a single institute 5 and National Cancer Database 7, which found that the response to chemotherapy and correlation of survival to pCR can be based on the breast and axillary nodes separately, or the combined treatment responses of breast and axillary nodes together, such as in RCB.

### Limitations

The retrospective analysis is one of the limitations of this study, as well as its shorter follow-up time. Meanwhile, not all HER2 positive cancers received trastuzumab-containing anti-HER2 therapy in our cohort. Although the use of trastuzumab for HER2-positive breast cancer had become standard of care since 2005, it was reimbursed as adjuvant or neoadjuvant therapy since 2010.

## Conclusions

This study demonstrates that N-pCR is highly correlated with B-pCR in all intrinsic subtypes of clinically node-positive breast cancer after NAC, and that the biologic heterogeneity of discrepancies between B-pCR and N-pCR rates to different treatment regimens needs further exploration. Our data did not support the omission of axillary surgery because worse DFS was found in patients with residual axillary node disease after NAC than those with residual breast disease.

## Supplementary Material

Supplementary figures and tables.

## Figures and Tables

**Table 1:** Patients' clinicopathological and treatment characteristics

Clinical features	No. of cases (%)
No. of patients	968
Age (years), median (IQR)*	49 (14.5)
Tumor size (cm), median (IQR)	4.2 (2.4)
Clinical T stage	
T1	39 (4.0)
T2	519 (53.7)
T3	192 (19.8)
T4	218 (22.5)
Clinical nodal status	
N1	543 (56.1)
N2	425 (43.9)
Histology	
Invasive ductal carcinoma	952 (98.4)
Mucinous carcinoma	2 (0.2)
Invasive lobular carcinoma	10 (1.0)
Invasive micropapillary carcinoma	4 (0.1)
Histologic grade	
1	69 (7.1)
2	371 (38.3)
3	440 (45.5)
Unknown	88 (9.1)
ER status	
Negative	383 (39.6)
Positive	585 (60.4)
PR status	
Negative	484 (50.0)
Positive	484 (50.0)
HER2 status	
Negative	530 (54.8)
Positive	438 (45.2)
Subtype	
HR(-) HER2(+)	216 (22.3)
HR(+) HER2(-)	382 (39.5)
HR(+) HER2(+)	222 (22.9)
HR(-) HER2(-)	148 (15.3)
Operation type	
Mastectomy	594 (61.4)
Breast conserving surgery	374 (38.6)
Neoadjuvant regimens	
Antracycline + taxane	471 (48.7)
Antracycline + taxane + cisplatin	189 (19.5)
Antracycline + taxane + herceptin	242 (25.0)
Antracycline + taxane + herceptin + perjeta	66 (6.8)
Adjuvant hormone therapy	
No	417 (43.1)
Yes	551 (56.9)
Adjuvant radiotherapy	
No	252 (26.0)
Yes	716 (74.0)

Abbreviations: IQR: interquartile range; ER: estrogen receptor; PR: progesterone receptor; HER2: human epidermal growth factor receptor 2.

**Table 2:** pCR and non-pCR rates of breast or axillary node after neoadjuvant chemotherapy (cN1,2)

Subtypes		B-pCR, (n=244) no. (%)	B-non pCR, (n=724) no. (%)	*p*
HR(-) HER2(+) (n=216)	N-pCR	85 (89.5)	48 (39.7)	<0.001
	N-non pCR	10 (10.5)	73 (60.3)	
HR(+) HER2(-) (n=382)	N-pCR	26 (89.7)	89 (25.2)	<0.001
	N-non pCR	3 (10.3)	264 (74.8)	
HR(+) HER2(+) (n=222)	N-pCR	61 (85.9)	65 (43.0)	<0.001
	N-non pCR	10 (14.1)	86 (57.0)	
HR(-) HER2(-) (n=148)	N-pCR	41 (83.7)	43 (43.4)	<0.001
	N-non pCR	8 (16.3)	56 (56.6)	

Abbreviations: pCR: pathological complete response; B-pCR: breast pathologic complete response; B-non pCR: breast pathologic non-complete response; HR: hormone receptor; HER2: human epidermal growth factor receptor 2; N-pCR: axillary node pathologic complete response; N-non pCR: axillary node pathologic non-complete response.

**Table 3:** Univariate and multivariate analysis of factors influencing the disease-free survival

Parameter	Univariate			Multivariate	
	Hazard ratio (95% CI)	*P* value		Hazard ratio (95% CI)	*P* value
Age (yrs)					
≦40	Reference				
41-60	0.892 (0.737-1.080)	0.241		-	
>60	0.907 (0.698-1.180)	0.468			
Clinical T stage					
T4	Reference			Reference	
T1	0.377 (0.236-0.601)	<0.001		0.602 (0.373-0.974)	0.039
T2	0.444 (0.373-0.529)	<0.001		0.603 (0.498-0.731)	<0.001
T3	0.555 (0.450-0.686)	<0.001		0.724 (0.581-0.901)	0.004
Clinical N stage					
N2	Reference			Reference	
N1	0.336 (0.284-0.397)	<0.001		0.473 (0.394-0.569)	<0.001
Operation type					
Mastectomy	Reference			Reference	
BCS	0.632 (0.530-0.755)	<0.001		1.102 (0.903-1.345)	0.340
Histologic grade					
3	Reference			Reference	
1	0.455 (0.311-0.665)	<0.001		0.468 (0.317-0.693)	<0.001
2	0.902 (0.766-1.062)	0.215		0.936 (0.789-1.110)	0.447
Subtypes					
HR(-) HER2(+)	Reference			Reference	
HR(+) HER2(-)	0.813 (0.667-0.990)	0.040		1.501 (1.091-2.066)	0.013
HR(+) HER2(+)	0.890 (0.708-1.118)	0.317		1.840 (1.356-2.496)	<0.001
HR(-) HER2(-)	0.992 (0.777-1.266)	0.949		0.962 (0.750-1.233)	0.758
Pathological response					
T-non pCR	Reference			Reference	
T-pCR	0.279 (0.177-0.439)	<0.001		0.350 (0.218-0.564)	<0.001
B-pCR	0.297 (0.196-0.448)	<0.001		0.368 (0.238-0.568)	<0.001
B-non pCR	0.809 (0.661-0.992)	0.041		0.899 (0.732-1.103)	0.307
N-pCR	0.364 (0.271-0.490)	<0.001		0.486 (0.354-0.668)	<0.001
N-non pCR	0.960 (0.777-1.186)	0.703		0.968 (0.783-1.196)	0.764
Hormone therapy					
No	Reference			Reference	
yes	0.684 (0.587-0.797)	<0.001		0.414 (0.315-0.544)	<0.001
Radiotherapy					
Yes	Reference			-	
No	0.925 (0.772-1.108)	0.399			

Abbreviations: CI: confidence interval; BCS: breast-conserving surgery; HR: hormone receptor; HER2: human epidermal growth factor receptor 2; T-non pCR: breast and axillary node pathologic non-complete response; T-pCR: breast and axillary node pathological complete response; B-pCR: breast pathologic complete response; B-non pCR: breast pathologic non-complete response; N-pCR: axillary node pathologic complete response; N-non pCR: axillary node pathologic non-complete response.

**Table 4:** Univariate and multivariate analysis of factors influencing the overall survival

Parameter	Univariate			Multivariate	
	Hazard ratio (95% CI)	*P* value		Hazard ratio (95% CI)	*P* value
Age (yrs)					
≦40	Reference				
41-60	0.897 (0.703-1.144)	0.380		-	
>60	0.987 (0.707-1.379)	0.939			
Clinical T stage					
T4	Reference			Reference	
T1	0.242 (0.119-0.492)	<0.001		0.460 (0.223-0.948)	0.035
T2	0.335 (0.267-0.421)	<0.001		0.477 (0.372-0.611)	<0.001
T3	0.512 (0.398-0.660)	<0.001		0.669 (0.513-0.871)	0.003
Clinical N stage					
N2	Reference			Reference	
N1	0.315 (0.252-0.395)	<0.001		0.533 (0.416-0.682)	<0.001
Operation type					
Mastectomy	Reference			Reference	
BCS	0.419 (0.320-0.549)	<0.001		0.776 (0.577-1.044)	0.094
Histologic grade					
3	Reference			Reference	
1	0.365 (0.216-0.616)	<0.001		0.397 (0.231-0.682)	<0.001
2	0.933 (0.759-1.147)	0.511		1.034 (0.833-1.283)	0.762
Subtype					
HR(-) HER2(+)	Reference			Reference	
HR(+) HER2(-)	0.782 (0.602-1.015)	0.065		1.693 (1.158-2.474)	0.007
HR(+) HER2(+)	0.988 (0.738-1.324)	0.937		2.194 (1.526-3.155)	<0.001
HR(-) HER2(-)	1.242 (0.919-1.679)	0.158		1.248 (0.920-1.692)	0.155
Pathological response					
T-non pCR	Reference			Reference	
T-PCR	0.255 (0.133-0.487)	<0.001		0.367 (0.188-0.718)	0.003
B-pCR	0.256 (0.141-0.465)	<0.001		0.359 (0.194-0.667)	0.001
B-non pCR	0.783 (0.608-1.009)	0.059		0.873 (0.677-1.127)	0.297
N-pCR	0.279 (0.181-0.430)	<0.001		0.411 (0.261-0.648)	<0.001
N-non pCR	0.954 (0.736-1.236)	0.720		0.965 (0.744-1.252)	0.789
Hormone therapy					
No	Reference			Reference	
Yes	0.575 (0.472-0.700)	<0.001		0.357 (0.259-0.492)	<0.001
Radiotherapy					
Yes	Reference			-	
No	1.065 (0.852-1.333)	0.579			

Abbreviations: CI: confidence interval; BCS: breast-conserving surgery; HR: hormone receptor; HER2: human epidermal growth factor receptor 2; T-non pCR: breast and axillary node pathologic non-complete response; T-pCR: breast and axillary node pathological complete response; B-pCR: breast pathologic complete response; B-non pCR: breast pathologic non-complete response; N-pCR: axillary node pathologic complete response; N-non pCR: axillary node pathologic non-complete response.

## Abbreviations

B-pCR: breast pathologic complete response
N-pCR: axillary node pathologic complete response
NAC: neoadjuvant chemotherapy
T-pCR: total pathologic complete response
N-non pCR: axillary node pathologic non-complete response
B-non pCR: breast pathologic non-complete response
T-non pCR: total pathologic non-complete response
HR: hormone receptor
HER2: human epidermal growth factor receptor 2
OS: overall survival
pCR: pathologic complete response
DFS: disease-free survival
cN+: clinically node-positive
cN0: clinically node-negative
IHC: immunohistochemistry
FISH: fluorescence in situ hybridization
IQRs: interquartile ranges
KM: Kaplan-Meier
CIs: confidence intervals
EFS: event-free survival
