# Supplementary material for: Discrepancy of Breast and Axillary Pathologic Complete Response and Outcomes in Different Subtypes of Node-positive Breast Cancer after Neoadjuvant Chemotherapy
Source: J Cancer. 2021 Jul 6;12(17):5365–74. doi: 10.7150/jca.62830 (PMC8317533; doi:10.7150/jca.62830)
Supplement: Supplementary file 1 — Supplementary figures and tables. [file jcav12p5365s1.pdf]

Supplementary Table 1A pCR and non pCR rates of breast or axillary node after neoadjuvant chemotherapy (cN1 only)

Subtypes		B-pCR (n=180) no. (%)	B-non pCR (n=363) no. (%)	*p*
HR(-) HER2(+) (n=121)	N-pCR	63 (92.6)	32 (60.4)	<0.001
	N-non pCR	5 (7.4)	21 (39.6)	
HR(+) HER2(-) (n=198)	N-pCR	23 (92.0)	78 (45.1)	<0.001
	N-non pCR	2 (8.0)	95 (54.9)	
HR(+) HER2(+) (n=137)	N-pCR	47 (92.2)	58 (67.4)	0.001
	N-non pCR	4 (7.8)	28 (32.6)	
HR(-) HER2(-) (n=87)	N-pCR	31 (86.1)	32 (62.7)	0.016
	N-non pCR	5 (13.9)	19 (37.3)	

Abbreviations: pCR: pathological complete response; B-pCR: breast pathologic complete response; B-non pCR: breast pathologic non-complete response; HR: hormone receptor; HER2: human epidermal growth factor receptor 2; N-pCR: axillary node pathologic complete response; N-non pCR: axillary node pathologic non-complete response.

Supplementary Table 1B pCR and non pCR rates of breast or axillary node after neoadjuvant chemotherapy (cN2 only)

Subtypes		B-pCR (n=64) no. (%)	B-non pCR (n=361) no. (%)	*p*
HR(-) HER2(+) (n=95)	N-pCR	22 (81.5)	16 (23.5)	<0.001
	N-non pCR	5 (18.5)	52 (76.5)	
HR(+) HER2(-) (n=184)	N-pCR	3 (75.0)	11 (6.1)	0.001
	N-non pCR	1 (25.0)	169 (93.9)	
HR(+) HER2(+) (n=85)	N-pCR	14 (70.0)	7 (10.8)	<0.001
	N-non pCR	6 (30.0)	58 (89.2)	
HR(-) HER2(-) (n=61)	N-pCR	10 (76.9)	11 (22.9)	0.001
	N-non pCR	3 (23.1)	37 (77.1)	

Abbreviations: pCR: pathological complete response; B-pCR: breast pathologic complete response; B-non pCR: breast pathologic non-complete response; HR: hormone receptor; HER2: human epidermal growth factor receptor 2; N-pCR: axillary node pathologic complete response; N-non pCR: axillary node pathologic non-complete response.

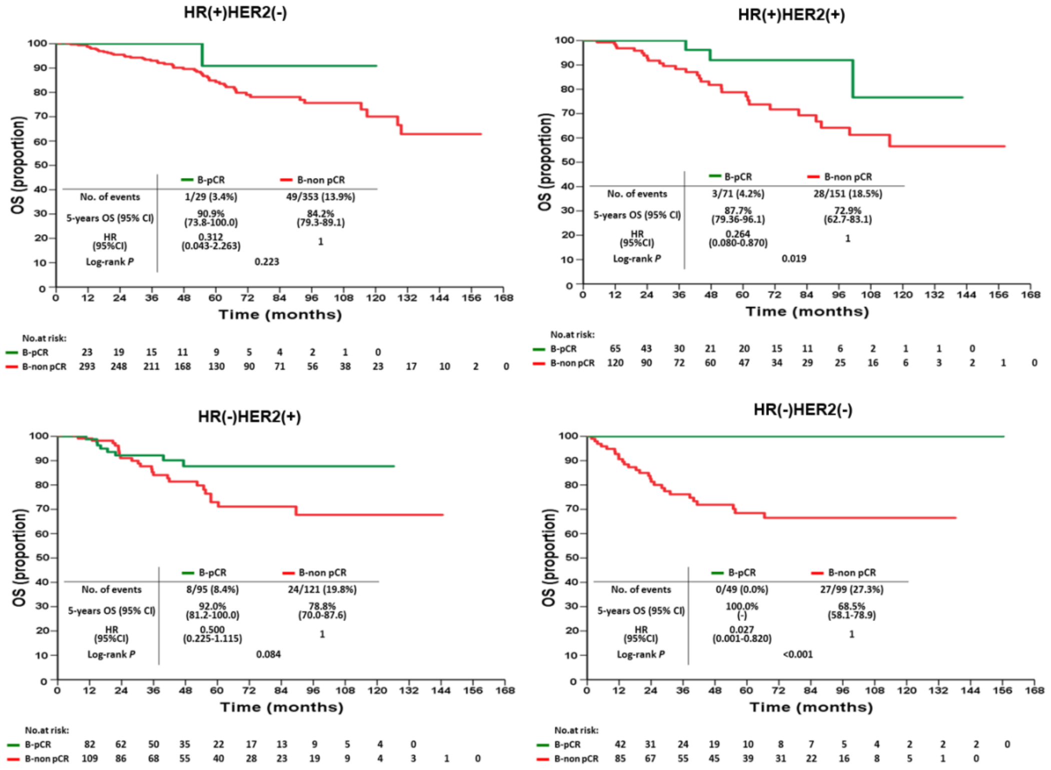

Supplementary Figure 1A Overall survival of B-pCR and B-non pCR patients after neoadjuvant chemotherapy by intrinsic subtypes

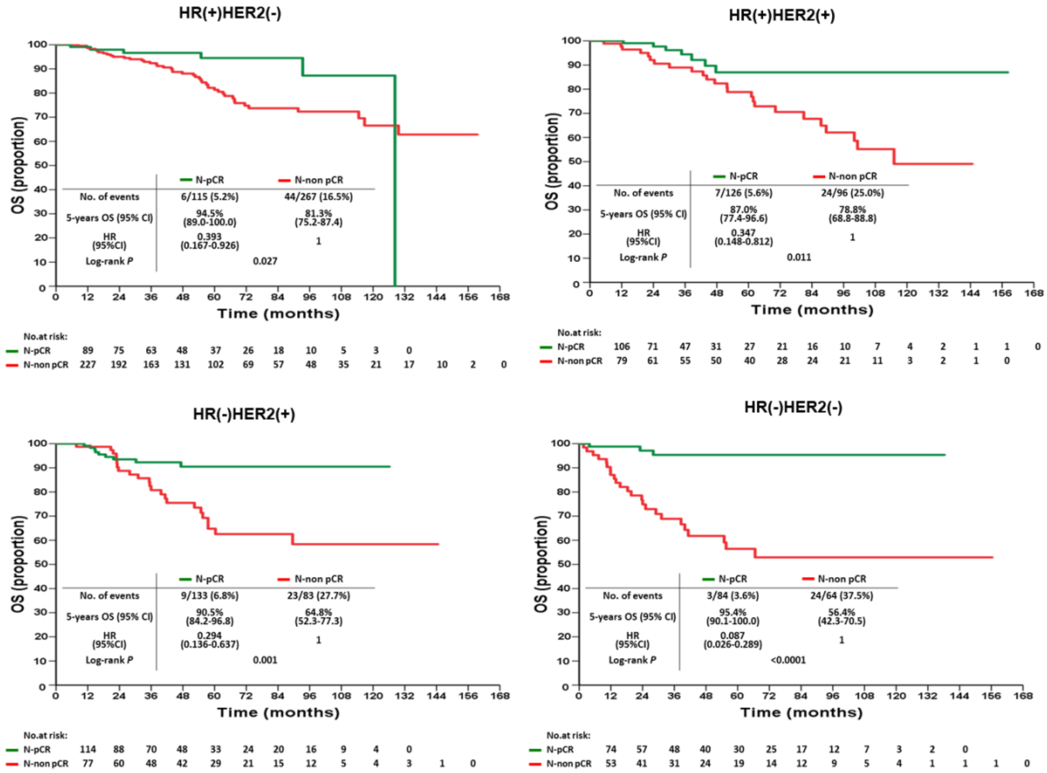

Supplementary Figure 1B Overall survival of N-pCR and N-non pCR patients after neoadjuvant chemotherapy by intrinsic subtypes
